# Characterisation of antibiotic prescriptions for acute respiratory tract infections in Danish general practice: a retrospective registry based cohort study

**DOI:** 10.1038/s41533-017-0037-7

**Published:** 2017-05-19

**Authors:** Rune Aabenhus, Malene Plejdrup Hansen, Laura Trolle Saust, Lars Bjerrum

**Affiliations:** 1 Section of General Practice and Research Unit for General Practice, Department of public Health, University of Copenhagen, Copenhagen, Denmark; 2 Research Unit for General Practice in Aalborg, Department of Clinical Medicine, Aalborg University, Aalborg, Denmark; 3 Department of Clinical Microbiology, Herlev Hospital, University of Copenhagen, Copenhagen, Denmark

## Abstract

Inappropriate use of antibiotics is contributing to the increasing rates of antimicrobial resistance. Several Danish guidelines on antibiotic prescribing for acute respiratory tract infections in general practice have been issued to promote rational prescribing of antibiotics, however it is unclear if these recommendations are followed. We aimed to characterise the pattern of antibiotic prescriptions for patients diagnosed with acute respiratory tract infections, by means of electronic prescriptions, labeled with clinical indications, from Danish general practice. Acute respiratory tract infections accounted for 456,532 antibiotic prescriptions issued between July 2012 and June 2013. Pneumonia was the most common indication with 178,354 prescriptions (39%), followed by acute tonsillitis (21%) and acute otitis media (19%). In total, penicillin V accounted for 58% of all prescriptions, followed by macrolides (18%) and amoxicillin (15%). The use of second-line agents increased with age for all indications, and comprised more than 40% of the prescriptions in patients aged >75 years. Women were more often prescribed antibiotics regardless of clinical indication. This is the first Danish study to characterise antibiotic prescription patterns for acute respiratory tract infections by data linkage of clinical indications. The findings confirm that penicillin V is the most commonly prescribed antibiotic agent for treatment of patients with an acute respiratory tract infection in Danish general practice. However, second-line agents like macrolides and amoxicillin with or without clavulanic acid are overused. Strategies to improve the quality of antibiotic prescribing especially for pneumonia, acute otitis media and acute rhinosinusitis are warranted.

## Respiratory tract infections: Tracking the overuse of antibiotics

Better adherence to guidelines for prescribing antibiotics for different respiratory tract infections are warranted in Danish general practice. The over-use of antibiotics, particularly so-called ‘second-line’ agents such as amoxicillin, increases resistance and may lead to a potentially catastrophic scenario where antibiotics are no longer effective. Exactly how widespread the over-use of antibiotics is for different infections, however, is not clear. Rune Aabenhus at the University of Copenhagen and co-workers analyzed primary care data regarding antibiotic prescriptions for acute respiratory tract infections including pneumonia and ear infections in Denmark. They found that penicillin V—the current recommended first-line drug in Scandinavian countries—accounted for 58 per cent of prescriptions, a figure which should be improved. Amoxicillin and macrolides were over-prescribed, particularly in elderly patients. The team also call for further analysis of prescriptions given by out-of-hours clinics.

## Introduction

Antimicrobial resistance rates have reached alarming levels and presently constitute a serious public health concern by threatening one of the most effective and mortality lowering interventions in modern medicine.^1^ Part of the solution to this problem includes minimizing overuse of antibiotics as they are directly linked to the development of antimicrobial resistance.^2^ As a consequence, it is imperative we make judicious use of the available antibiotics in order to maintain their effectiveness in the years ahead.

Acute respiratory tract infections are common reasons for consulting in general practice and assumed responsible for more than 60% of the antibiotic use in this setting.^3^ But the effect of antibiotic treatment, when pneumonia is not suspected, is at best moderate,^4–6^ indicating that a large amount of antibiotic prescriptions are in fact inappropriate and confer no net benefit for the patient. Strategies, such as guidelines on rational antibiotic use, as well as educational and decision support systems are applied to improve antibiotic prescribing. However, the implementation of these strategies into daily clinical routine can be difficult and daily practice is far from optimal^7, 8^ in turn augmenting the risk of inappropriate prescribing both in regard to quantity (e.g., overprescribing) and quality (e.g., non-optimal choice of antibiotic).^9^

As antibiotic resistance rates are low in Denmark^10^ National Guidelines^11^ on antibiotic prescribing for acute respiratory tract infections recommend narrow-spectrum penicillin (penicillin V) as first-line agent for acute respiratory infections, except acute exacerbations of chronic obstructive pulmonary disease (AECOPD) where the recommendation was amoxicillin with clavulanic acid (co-amoxicillin).

In Denmark, previous attempts to assess current prescribing practice for specific infections have been based on small subsets of general practitioners that volunteered to participate for a limited time.^12^

Surveillance of antibiotic prescribing and consumption is a central element in containing the increasing antimicrobial resistance.^9, 13^ Patterns of antibiotic prescriptions, particularly when linked to clinical indications and patient demographics, can be helpful in determining appropriateness of the issued prescribing: lately results from the UK and the Netherlands have successfully applied data linkage to characterise prescribing of flucloxacillin and analyze trends in prescribing for sore throat, colds, and acute cough.^14–16^

To promote rational antibiotic use in Denmark, and study the link between clinical indications and the associated antibiotic prescribing, an electronic system with mandatory data entry of clinical indications for antibiotic prescribing was introduced in 2011.

We aimed to characterise the pattern of antibiotic prescriptions for acute respiratory tract infections, by means of clinical indications from electronic prescriptions, in regard to national guidelines on antibiotic prescribing in Danish general practice.

## Results

### Clinical indications for antibiotic prescribing

A total of 2.4 million systemic antibiotic prescriptions (ATC J01) corresponding to 424 treatments per 1000 inhabitants were issued in the 1-year study period. A clinical indication was assigned in 1.6 million prescriptions, however about 25% (416,355) of the prescriptions did not include a specific clinical indication, but only stated e.g. “against infection”. Acute respiratory tract infections accounted for 456,532 prescriptions, of which pneumonia accounted for 178,354 (39%), followed by tonsillitis (*N* = 96.549; 21%), acute otitis media (AOM, *N* = 87.064; 19%), acute rhinosinusitis (*N* = 81.662; 18%), AECOPD (*N* = 9967; 2%), and acute bronchitis (*N* = 2.935; 1%) (Table 1).

**Table 1:** Antibiotic use pattern for acute respiratory tract infections in Danish general practice

Clinical indication	Acute otitis media	Acute tonsillitis	Acute rhinosinusitits	Acute bronchitis	Pneumonia	Acute exacerbation of COPD^a^	Total acute respiratory tract infection
Penicillin V J01CE N (%)	47.612 (54.7)	83.127 (86.1)	53.097 (65.0)	11 (0.4)	78.909 (44.2)	633 (6.4)	263.389 (57.7)
Amoxicillin J01CA N (%)	31.479 (36.2)	6.211 (6.4)	7.925 (9.7)	<6 (<0.2)	23.325 (13.1)	<6 (0.2)	68.940 (15.1)
Co-amoxicillin J01CR N (%)	2.340 (2.7)	1.200 (1.2)	2.745 (3.4)	<6 (<0.2)	24.830 (13.9)	8.521 (85.5)	39.636 (8.7)
Macrolide^b^ J01FA N (%)	5.604 (6.4)	6.010 (6.2)	17.484 (21.4)	1.636 (55.7)	48.630 (27.3)	672 (6.7)	80.036 (17.5)
Quinolone J01MA N (%)	26 (<0.2)	<6 (<0.2)	40 (<0.2)	<6 (<0.2)	2.581 (1.4)	120 (1.2)	2.768 (0.6)
Tetracycline J01AA N (%)	<6 (<0.2)	<6 (<0.2)	370 (0.5)	1.282 (43.8)	<6 (<0.0)	<6 (<0.2)	1.654 (0.4)
Other J01^c^ N (%)	<6 (<0.2)	<6 (<0.2)	<6 (<0.2)	<6 (<0.2)	70 (0.0)	21 (0.2)	95 (0.0)
Total N (%)	87.064 (100)	96.549 (100)	81.662 (100)	2.935 (100)	178.354 (100)	9.967 (100)	456.532 (100)

Antibiotic use was regarded as rational and in accordance with national guidelines, when minimum 80% of treatments were the recommended first-line agent^44^

According to Danish standards for data protection, counts <6 for individual prescribing are not specified

^a^ COPD chronic obstructive pulmonary disease

^b^ Lincoamides account for 0,2% of total macrolide use

^c^ Other J01 Cephalosporin (J01D) 21%; beta-lactamase-resistant penicillin (J01CF) 77%; sulfonamides (J01EB02) 1,1%; nitrofurantoin (J01XE01) 1,1%

Women were more often prescribed antibiotics regardless of clinical indication (female:male ratio 1.33; 95% confidence interval (CI) 1.32–1.34). Notably, women were prescribed antibiotics twice as often as men (female:male ratio 2.18; 95% CI 2.14–2.21) in acute rhinosinusitis.

**Fig. 1:**
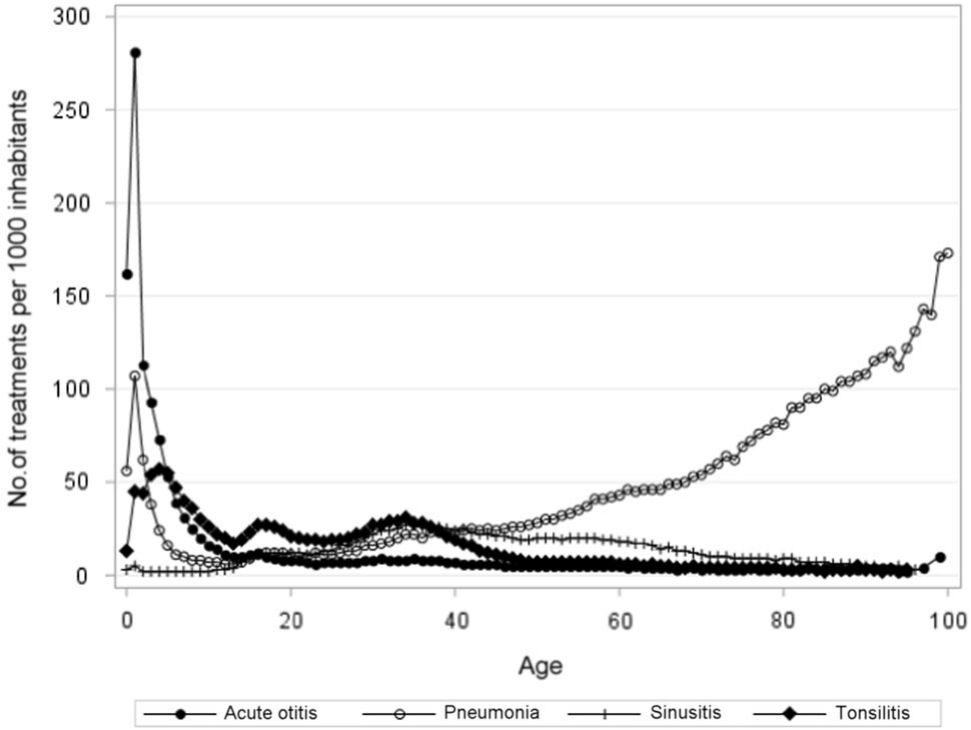
Antibiotic use for patients with acute respiratory tract infections by age

Marked variations were found in relation to age (Fig. 1). Prescribing for AOM and pneumonia was high in children under 5 years of age (total exposure for acute respiratory infection was 244 treatments per 1000 inhabitants less than 5 years). Middle-aged adults were more commonly prescribed antibiotics for acute rhinosinusitis and tonsillitis, yet exposure in this group (15–74 years) was low with a range between 65 to 80 treatments per 1000 inhabitants. The clinical indication in older adults (>75 years) was predominantly pneumonia and exposure to antibiotics for respiratory tract infection was high (264 treatments per 1000 inhabitants).

### Patterns of antibiotic prescriptions

**Fig. 2:**
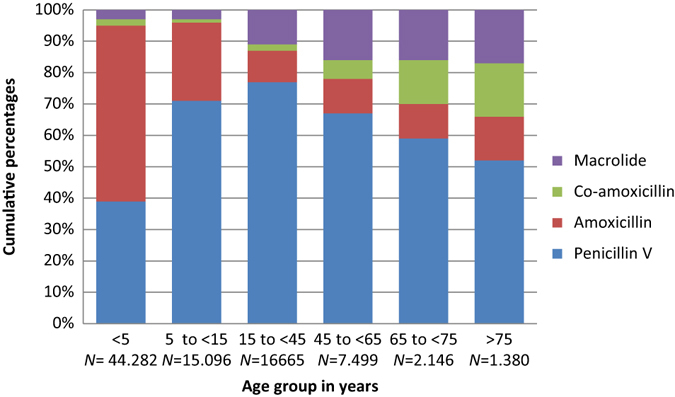
Acute otitis media. Prescribing pattern by age group (*N* = 87.064)

**Fig. 3:**
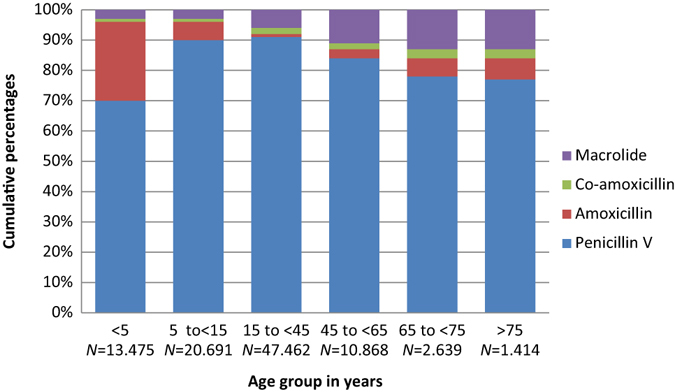
Acute tonsillitis. Prescribing pattern by age group (*N* = 95.459)

**Fig. 4:**
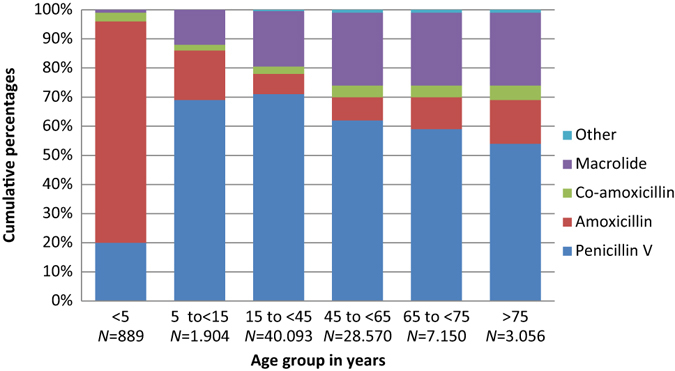
Acute rhinosinusitis. Prescribing pattern by age group (*N* = 81.662)

**Fig. 5:**
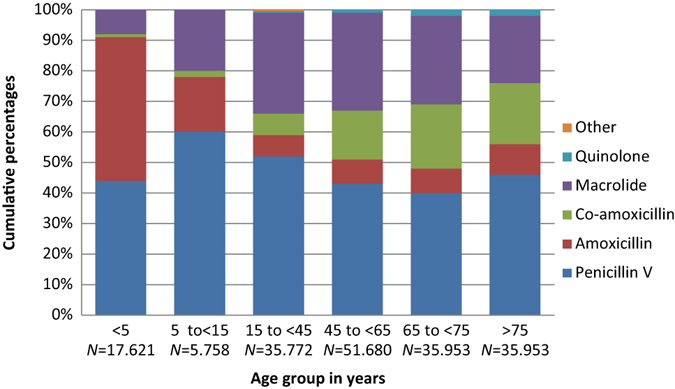
Pneumonia. Prescribing pattern by age group (*N* = 178.354)

In total, penicillin V accounted for 57.7% of all prescriptions; macrolides second (18%) and amoxicillin third (15%). Quinolone and tetracycline use were low. Antibiotic use patterns differed between the individual clinical indications (Table 1). Penicillin V use was highest in acute tonsillitis (86%), in contrast to acute bronchitis where only 0.4% of prescriptions were penicillin V. Macrolide use was highest in acute bronchitis (55.7%) and pneumonia (27.3%). Co-amoxicillin comprised 86% of the total antibiotic use in patients diagnosed with AECOPD, but was also prescribed to one in seven diagnosed with pneumonia. Use of amoxicillin was higher than penicillin V among children aged less than 5 years: AOM (56% vs. 39%); acute rhinosinusitis (75% vs. 20%); pneumonia (48% vs. 43%) but not in acute tonsillitis (26% vs. 70%) (Figs. 2–5).

From 5 years and up, the use of second-line agents increased with age for all respiratory indications, and more than 50% of the prescriptions comprised second-line agents in patients aged >75 years. The clinical indication acute tonsillitis was the only indication with a relatively high proportion of penicillin V among the elderly (Figs. 2–5).

### Antibiotic prescriptions in regard to guidelines on antibiotic prescribing

Overall penicillin V use was less than expected according to our predetermined criteria of at least 80% first-line agents (Table 1). However, in children under the age of 5 years, more than 90% of prescriptions comprised penicillin V or amoxicillin.

We found that 30.0% of prescriptions for AOM were administered to children younger than 2 years of age, and 50.9% before age 5 years (Figs. 1 and 2). In acute tonsillitis, prescribing in toddlers younger than 3 years of age occurred in 6.6% (Figs. 1 and 3). Similarly, antibiotics were infrequently prescribed in children with acute rhinosinusitis under the age of 5 years (1.1%) and under the age of 15 years (3.4%) (Figs. 1 and 4).

## Discussion

### Main findings

This is the first study to link clinical indications to antibiotic prescribing for acute respiratory tract infections in Danish general practice.

Based on all prescriptions, pneumonia was the most common clinical indication (39%), followed by acute tonsillitis (21%), AOM (19%), and acute rhinosinusitis (18%). The frequency of the clinical indications showed pronounced associations with age. We found that penicillin V was the most commonly used antibiotic agent (58%), macrolides second (18%), and amoxicillin third (15%).

The relative low use of penicillin V is not in line with guidelines recommendations on acute respiratory tract infections (except AECOPD) indicating that current prescribing could still be improved in order to better adhere to Danish guidelines on antibiotic use. Importantly, we found that the use of second-line agents including critical macrolides that belong to the WHO-defined group of antibiotics of the highest priority^17^ increased, especially in the older age group. Further efforts are needed to promote rational antibiotic use in patients with acute respiratory tract infection, particularly for AOM, acute rhinosinusitis, and pneumonia.

### Interpretation of findings in relation to previously published work

Few non-Scandinavian countries recommend penicillin V as first-line agent and the European Society for Clinical Microbiology and Infectious Disease recommend amoxicillin or a tetracycline in lower respiratory tract infections.^18^ No clear evidence has pointed to a specific antibiotic agent as most effective in empirical antibiotic therapy.^19^ As most serious acute respiratory infections are related to *pneumococci*, penicillin V may (in the absence of high resistance rates) be regarded a rational choice due to its high activity against gram positive cocci (minimal inhibitory concentration 0.06 µg/ml) ^20^ and few side effects.

In young children, who for the most part will use syrup or other liquid formulations of antibiotics, the high use of amoxicillin is also reported in studies from Norway^21^ and this is most probable related to the superior taste of amoxicillin compared to the first-line agent penicillin V. Accordingly, it may be reasonable to prescribe amoxicillin to secure compliance with treatment,^22^ also as recent studies have suggested that resistance selection by amoxicillin is mostly short-lived (less than 1 month).^23^ However the long-term effects and increased risk of resistance^24^ calls for caution, and antibiotic prescribing should be targeted to patients with a high chance of benefit.^5, 25^

Antibiotic use for patients diagnosed with acute tonsillitis was predominantly penicillin V (86%),which may be perceived as in line with national recommendations.^11, 26^ Furthermore, less than 6% of the antibiotic prescriptions for acute tonsillitis were administered in children under 3 years, a group having a low a priori risk of strep throat.^27^ In Dutch children, the use of first-line treatment with penicillin V in acute tonsillitis was 60%.^8^ Most Danish general practitioners (GPs) use point-of-care tests to swab for the presence of *Streptococcus* group A antigen as part of the clinical management of sore throat. Identifying a causable pathogen may lead to choosing a relevant narrow-spectrum agent such as penicillin V.

Today, AOM is mostly perceived as a self-limiting disease in children older than 2 years of age and the modest benefit of antibiotic treatment has been established.^6^ Still two-thirds of all prescriptions are given in children older than 2 years of age and half in patients aged more than 5 years. This finding indicates a possible overuse of antibiotics in patients diagnosed with AOM, which is a common reason for antibiotic use in children.^16^

In contrast, age-stratified antibiotic prescribing in patients diagnosed with acute rhinosinusitis appeared meaningful as only 3% of prescriptions were issued in children under 15 years of age. Children do not have fully developed sinuses until adolescence. However, it is important not to miss this diagnosis due to the risk of orbital or endo-cranial complications, especially in young children under 6 years of age.^28^ In adults, the antibiotic use in acute sinusitis has previously been assessed as inappropriate as 60% have had less than 5 days of symptoms prior to presentation and only 30% presented with fever.^29^

The clinical indication “pneumonia” made up 39% of all antibiotic prescriptions for acute respiratory tract infections. In 2012, there were 30,000 hospital admissions with pneumonia and 1555 patients died (www.statistikbanken.dk). It has previously been estimated that the number of patients seen in general practice was up to five times greater, which mounts to roughly the number of clinical indications “pneumonia” the same year (178.354). This number may however be affected by the limited available indications in the electronic prescription system (no clinical indication exists for cold or cough) or due to diagnostic drift, i.e., that the doctor tends to justify a prescription by labeling it”pneumonia”.^30^ Secondly, GPs may have entered a free text indication or selected an unspecific term “infection”, as such not permitting a detailed analysis of the prescribing. Lastly, some cases of mild pneumonia may not have been prescribed antibiotics or perhaps the patient never filled the prescription (primary non-compliance in Denmark is 6.5% for antibiotic scripts).^31^ This may indeed be the case as results from the European project Happy Audit^32^ found 17% of acute respiratory tract infections to be acute bronchitis with an associated antibiotic prescribing rate of 35%. The striking differences to the national figures based on the clinical indications we present in this paper is problematic, as the current system of electronic prescriptions may not capture a sufficiently detailed picture of antibiotic prescribing.

Generally, we observed a trend towards increasing prescribing of second-line agents in older age groups. A macrolide is recommended in case of penicillin allergy; however it does not seem plausible to ascribe all of observed broad-spectrum use to penicillin V allergy or penicillin resistant bacteria detected by microbiological testing. The exact reason for the increase is not deducible from the present data, but factors involved may include uncertainties in management and diagnosis, patient preferences including ease of administration and a difference in the suspected microbiological etiology of acute respiratory tract infections. Of note, no mycoplasma epidemic was observed in the winter 2012–2013. Still one in four patients with pneumonia was prescribed a macrolide. Similar findings concerning overuse of second-line agents have been presented from the Netherlands.^33^

Prescribing of other critical antibiotics of the highest priority,^17^ namely 3th and 4th generation cephalosporins and quinolones was low (0,1 and 0.6%). These findings are in line with the Danish recommendations stating that these agents should only be prescribed following microbiological identification and known susceptibility to treatment.^34^ Moxifloxacin was the main quinolone prescribed for AECOPD and acute rhinosinusitis, while ciprofloxacin was issued for patients diagnosed with pneumonia or AOM.

The broader the antibiotic spectrum an agent has, the larger the effect on the intestinal flora. Penicillin V is considered a narrow-spectrum antibiotic, but most second-line agents have a broader spectrum of antibacterial activity. Avoidance of inappropriate use of second-line agents is a requirement to limit harm to the patients’ normal flora and minimize risk of developing antibiotic resistance in common bacteria.

### Strengths and limitations of the study

The electronic data collection ensured complete and valid routine data, from GPs not aware of the investigation, making the results generalizable to other settings.

We did not have data to assess the specific consultation rates for acute respiratory tract infections. As a result, we are unable to compare data on proportion of consultations for acute respiratory tract infection that resulted in an antibiotic prescription.^15^ Also, the number of treatments per age group provided should be interpreted with caution as missing and unspecific indications are not included. However, the total antibiotic use of 424 treatments per 1000 inhabitants per year can be compared to prescriptions rates from a Dutch study^33^ of 45 primary practices with a prescribing rate of 300 treatments per 1000 patient years. The Netherlands is a low antibiotic prescribing country and furthermore out-of-hours use was not included.

A substantial number of prescriptions were not available for analysis due to either no clinical indication present on the electronic prescriptions or non-specific indications hampering the precise assessment of incidences of specific acute respiratory infections. However, the remaining large number of data can provide a valid specification of the antibiotic use pattern for selected clinical indications, as we did not find evidence of a systematic difference regarding patient or provider age and sex in the overall antibiotic use between scripts with no indications or unspecific indications vs. scripts with specific indications (data not shown).

A number of clinical indications may be missing from telephone or handwritten prescriptions. We have no methods to assess this amount, but we believe this number is relatively small and with no substantial impact on the antibiotic use pattern.

It may be argued, those GPs who are not up-to-date or who do not want to be part of surveillance systems could be underrepresented or not coding in a meaningful way. The clinical indication functionality on electronic prescriptions is still quite new and not all GPs may find it intuitive, easy to use or relevant. A GP could just use the first indication from the drop down menu throughout the day. This would threaten the validity of the assigned clinical indications and make the antibiotic use pattern less precise. We believe this problem is virtually impossible to eliminate, but future studies should be undertaken to improve understanding of prescriptions with missing and non-specific clinical indications.

### Implications for future research, policy and practice

Future studies would benefit from differentiating between daytime and out-of-hours antibiotic use as Huibers et al. have shown that out-of-hours antibiotic use was high and often second-line agents were issued, especially among patients >60 years of age.^35^

Assessing rational antibiotic prescribing is challenging as no formal quality indicators have been formally developed and validated in a Danish setting.^36, 37^ A quality indicator is defined as a specific and measurable instrument that can be used to assess the quality of care provided.^38^ For optimal impact and validity context, specific quality indicators should be developed and implemented. This process is presently ongoing in Denmark. The systematic implementation of such valid quality indicators may be beneficial to future patients by assisting individual clinics striving to improve care as well as to monitor national surveillance targets on antibiotic use.^39^

The number of unspecific indications is high and an effort to reduce this group is warranted, bearing in mind that not all cases issued a prescription may present with a clear site of infection at the time of consultation. Possible changes might include grouping of selected clinical indications, such as lower or upper acute respiratory tract infection, and providing more options for symptoms or complaints in the clinical indications such as cold and cough. To more accurately reflect the clinical indications for which antibiotics are prescribed and thus optimize understanding changes in antibiotic use, a more direct link from medical records into the electronic prescribing system is warranted.

### Conclusions

This study confirms that penicillin V is the most commonly prescribed antibiotic agent for treatment of patients with an acute respiratory tract infection in Danish general practice. However, the high prescribing rate of second-line agents, like macrolides and extended-spectrum penicillins, could be improved significantly. Future initiatives to promote rational prescribing of antibiotics should address specific clinical indications like AOM, acute rhinosinusitis, and pneumonia.

## Methods

### Data sources

All Danish citizens who redeemed an antibiotic prescription (ATC J01, see below for details) between July 1st 2012 to June 31st 2013 were included. This 1-year study period was chosen to ensure a coherent winter season with its corresponding influenza peak.^40^

National data on use of antibiotic therapy in Denmark was extracted from the Danish National Prescription Database.^41^ This registry contains complete information on all prescriptions redeemed by Danish residents at outpatient pharmacies from January 1st 1995 onwards. Antibiotics are only available by prescription and purchased solely in pharmacies in Denmark. For each redeemed prescription, the registry contains information on the following variables relevant to this study: provider practice identification number (encrypted), type of antibiotic, patient age and gender, as well as clinical indication.

Clinical indications are provided by the electronic prescription system. As such they are not directly linked to the International Classification of Primary health Care (ICPC-2) coding system used in the GPs medical records. The GP must select an appropriate clinical indication from a pre specified drop down list or enter a free text indication prior to signing of an antibiotic.

Antibiotic agents were classified according to the anatomical therapeutic chemical (ATC) index (J01 antibacterial agents for systemic use), down to 5th level; chemical substance.^42^

The Danish Civil Registration System^43^ contains data on all Danish residents through an individual identifier (encrypted), which allowed us to keep track of all study subjects.

General practice providers were identified by the unique identifiers for general practitioners (encrypted), obtained from Statens Serum Institute, to distinguish GP prescriptions from the entire primary care sector, which also includes other medical providers such as ear-nose-throat specialist, dentist etc.^40^ All information on patient and prescriber level was encrypted.

Statistikbanken.dk, a service from the Statistics Danmark, was used to determine population size in total and the included age groups.

During the study period (2012–2013), the Danish national guideline^11^ on antibiotic use recommended penicillin V as empirical first choice for AOM, acute tonsillitis, acute rhinosinusitis, and pneumonia. In case of penicillin allergy, a macrolide was recommended. The recommendation for AECOPD was co-amoxicillin. In case of penicillin allergy, a tetracycline was recommended. Uncomplicated acute bronchitis is considered a self-limiting disease and no antibiotic is recommended.

We assumed that recommended empirical first-line treatment should account for at least 80% of prescriptions in order to adhere to guidelines.^44^

No specific guidance regarding diagnostics were included, however a general recommendation regarding use of C-reactive protein was adopted in general practice (the C-reactive protein speedometer).^45^

### Analyses

A dataset consisting of clinical indications and antibiotic prescriptions was created by merging datasets by their unique practice identification numbers.The following six age groups for each specific indication were used;^40^ <5 years; 5–14 years; 15–44 years; 45–64 years; 65–74 years, 75+ years.

When assessing the clinical indications for antibiotic prescribing, we chose to include the full range of acute respiratory tract infections; all R codes in the International Classification of Primary Care, second edition.^46^ As a result, both upper and lower respiratory tract infections were included. Furthermore we chose to include AOM to the group of respiratory tract infections in primary care. This was due to the fact that the clinical picture of AOM rarely presents as an isolated disease entity, but most often is accompanied by additional common cold symptoms.

The clinical indicators on the most common acute respiratory tract infections in primary care were grouped and analyzed: AOM, acute tonsillitis, acute rhinosinusitis, acute bronchitis, pneumonia, and AECOPD. Descriptive analyses of antibiotic use were performed, such as percentages, cumulated percentages, no. of treatments per 1000 inhabitants for each acute respiratory tract infection.

In addition, the accordance of GPs’ antibiotic management of patients with acute respiratory tract infections was assessed by comparing to national guidelines.^11^ Danish national guidelines on antibiotic use recommends narrow-spectrum penicillin (penicillin V) as first-line agent for most respiratory cases, except AECOPD, where the recommendation in 2012–2013 was co-amoxicillin. All calculations were done in SAS version 9.3 (Cary, NC, USA).

### Ethics

The study was approved by the Danish Data Protection Agency (J.nr. 2012-41-0159). According to Danish law, no approval from an ethics committee is required for strictly registry based studies.

### Data availability

All relevant data are available from the authors, however Danish law on data protection prohibits access to raw data.
